# Temperature, but not excess of glycogen, regulates “in vitro” AMPK activity in muscle samples of steer carcasses

**DOI:** 10.1371/journal.pone.0229480

**Published:** 2021-01-28

**Authors:** Pablo Strobel, Alex Galaz, Franz Villaroel-Espíndola, Ariel Apaoblaza, Juan Carlos Slebe, Nancy Jerez-Timaure, Carmen Gallo, Alfredo Ramírez-Reveco

**Affiliations:** 1 Instituto de Ciencia Animal, Facultad de Ciencias Veterinarias, Universidad Austral de Chile, Valdivia, Chile; 2 Instituto de Bioquímica y Microbiología, Facultad de Ciencias, Universidad Austral de Chile, Valdivia, Chile; 3 Laboratorio Medicina Traslacional, Fundación Arturo López Pérez Cancer Center, Santiago, Chile; Universidade Federal de Viçosa, BRAZIL

## Abstract

Postmortem muscle temperature affects the rate of pH decline in a linear manner from 37.5°C to 0–2°C. The pH decline is correlated with the enzymatic degradation of glycogen to lactate and this process includes the metabolic coupling between glycogenolysis and glycolysis, and that are strongly upregulated by the AMPK. In this study, we used 12 samples previously characterized by have different muscle glycogen concentration, lactate and AMPK activity, selected from 38 steers that produced high final pH (>5.9) and normal final pH (<5.8) carcasses at 24 h postmortem. Moreover, we evaluated changes in the AMPK activity in samples from both categories incubated at 37, 25, 17 and 5°C and supplemented with exogenous glycogen. Finally, we analysed if there were structural differences between polymers from both categories. Our results showed that “in vitro” enzymatic AMPK activity evaluated at both 0.5 or 24 h was greater in samples from normal then high pH categories (p <0.01), and in all temperature of incubation analysed (17, 25 and 37°C). For other hand, a greater AMPK activity were obtained in samples incubated at 17 that 25 or 37°C, in normal carcasses at both 0.5 or 24 h (p < 0.01), as also in samples from carcasses categorized as high pH, but at 24 h (p < 0.05). Interestingly, AMPK activity was totally abolished at 5°C, independent of final pH category of carcasses, and was confirmed that the incubation temperature at which the maximum activity was obtained (p < 0.01), at least in carcasses with a normal pH is at 17°C. The enzymatic AMPK activity did not change in relation to excess glycogen (p > 0.05) and we did not detect structural differences in the polymers present in samples from both categories (p > 0.05), suggesting that postmortem AMPK activity may be highly sensitive to temperature and not to *in vitro* changes in glycogen concentration (p > 0.05). Our results allow concluding that normal concentrations of muscle glycogen immediately at the time of slaughter (0.5 h) and an adequate cooling managing of carcasses are relevant to let an efficient glycogenolytic/glycolytic flow required for lactate accumulation and pH decline, through the postmortem AMPK signalling pathway.

## Introduction

In Chile and in other countries with extensive pasture fattening systems, the high pH of meat is still an important meat quality problem [1–3]. Studies in Chile have shown that the incidence of high pH meat varies between 17 and 40% on a yearly basis [1] and the problem causes significant losses to the meat industry [4]. There are several studies [5–9] that have addressed the relationship between the adequate decline of pH post-slaughter with normal activity and/or expression of metabolic enzymes. Special attention has been focus on the Adenosine Monophosphate kinase (AMPK) enzyme. The main function of the AMPK is the coordination of metabolic processes (anabolic and catabolic) in various organs or tissues including brain, pancreas, liver, heart, skeletal muscle and adipose tissue [10, 11]. Hence, the consequences of AMPK activation are accompanied by an acute regulation of energy metabolism and chronic changes in gene expression, and these changes are accompanied by phosphorylation of several effector proteins that include biosynthetic enzymes, transporters, transcription factors, ion channels and signaling proteins of the cell cycle [12–14].

Nerve and muscle functions in most vertebrates are seriously affected by freezing, to which three stress components have been associated: changes in the Intra/extracellular ions, ischemia, and low temperature [15–18]. Environmental stress can also modify the AMPK activity. Hypoxia and hypothermia in increase the AMPK phosphorylation level and AMPK activity in lower vertebrates [19]. Effectively, winter survival of hundreds of species depends on freeze tolerance and one of these species (*Rana sylvatica*) revealed that during in vivo freezing, AMPK enzyme increases its activity [20]. This suggests that temperature has an important role in AMPK activity that would facilitate the best adaptation to hypothermia, freezing and thawing.

Some studies have clearly shown that in postmortem muscles there is a strong correlation between AMPK activation and pH decline, indicating that AMPK regulates glycolysis in postmortem muscles [8, 21–23]. In porcine *longissimus* muscle, an earlier and faster activation of AMPK is responsible for lower pH and higher lactic acid levels in PSE (pale, soft exudative) meat [8]. In bovine *longissimus* muscle studies, we have shown that AMPK activity was four times greater in carcasses that experienced a normal decrease in pH at 24 h, versus those that experienced a low decrease in pH in the same time and condition. This pH after decline was conditioned by the initial reserve levels of glycogen at slaughter and by efficient glycogenolytic/glycolytic flow in postmortem muscle [5]. Finally, there are insufficient data about the changes in postmortem muscle AMPK activity associated to low temperatures and/or to postmortem glycogen level. The aim of this study was to determine changes in postmortem AMPK activity due to temperature decline and to excess of glycogen level in muscle samples from steer carcasses with normal final pH (<5.8) and high final pH (>5.9).

## Material and methods

### Ethics approvals

The postmortem samples used were obtained from animals previously transported and handled following the guidelines defined by the slaughterhouse and the regulations of the Bioethics Committee for use of animals in research at Universidad Austral de Chile (Certificate N° 34–2011).

### Animals, slaughter, processing, and categorization of samples

In this study, we used 12 samples previously characterized by have different muscle glycogen concentration, lactate and AMPK activity, selected from 38 steers that produced high final pH (>5.9) and normal final pH (<5.8) carcasses at 24 h postmortem.

Animals were under the same fattening systems (mainly pasture and silage) and had similar weights and fatness leves (visual appretiation). The antemortem and postmortem management as well as sampling from all beef carcasses are described in Apaoblaza et al., [5]. Samples of approx. 1 g were taken from the *Longissimus thoracis* (LT) muscle of the carcasses (at the level of the 10th rib) using a Bergström biopsy needle at 0.5 h postmortem (T0). After 24 h of chilling (0–4°C), the final temperature (T24) and pH (pH 24) were measured, and a second muscle sample was taken from all carcasses at the same place. These samples (T0 and T24) were placed in cryotubes, and immediately frozen in liquid nitrogen (−196°C) and maintained in an ultra-freezer (−80°C). Six samples that had a pH 24 > 5.9 (high pH, n = 6) and six samples that had a pH 24 < 5.8 (normal pH, n = 6) were selected for this study.

### Temperature kinetic in carcasses

To establish the kinetic decline of temperature and pH (initial and final) of the carcasses in the cooling room from T0 to T24 h (0 to 1440 minutes), a thermocouple/ electrode penetration probe with data logger (PCE 228 M, Spain) was connected to each carcass.

### Muscle glycogen concentration (MGC) determination

This analysis considered a glycogen purification and enzyme digestion method to obtain glucose [24]. Briefly, 400 μl of 30% KOH were added to 100 mg of tissue, followed by incubation at 100°C for 15 min with agitation. A 50 μl sample of each homogenate were placed on a 1 × 1 cm Whatman 31 ET filter paper. A calibration curve with glycogen (2.4 mg/ml) was prepared. Glycogen was precipitated with 66% cold ethanol for 10 min while stirring. Then, two washes were performed with 66% cold ethanol and the papers were dried to remove ethanol. Afterwards, 1 ml of amyloglucosidase (Sigma, 10113) solution 0.5 mg/ml in 400 mM sodium acetate buffer pH 4.8 was added and was incubated for 2 h at 37°C in a stirred thermoregulated bath. Afterwards, 50 μl were extracted from each tube for the determination of glucose using the Glucose PAP liquiform kit (Labtest, Diagnóstica S.A.) and spectrophotometric assay by glucose oxidase and Trinder reaction, in which the product is a quinoneimine dye, which absorbs maximally at 505 nm [25]. Finally, it was taken to initial glycogen concentration through stoichiometry.

### Lactate (LA) determination

Lactate concentration was determined by using the lactate oxidase method (Lactate Liquiform, Labtest Diagnóstica S.A.), according to Siqueira et al., [26]. Briefly, 100 mg of muscle samples frozen in liquid nitrogen were homogenized in 20 mM citrate–50 mM phosphate buffer, EDTA 2.5mg/ml pH 6.8. An aliquot of the homogenate was used to determine the concentration of LA.

### AMPK activity assay

From each sample, 200 mg of muscle was powdered (liquid nitrogen) and homogenized in Ultra-Turrax T-10 (IKA-Werke GMBH, Germany) with an ice-cold buffer containing 500 μl lysis solution, 137 mM NaCl, 1 mM MgCl2,1% NP-40, 10% glycerol, 2 mM PMSF, 10 mM sodium pyrophosphate, 2.5 mM EDTA, 10 μg/ml Aprotinin, 10 μg g/ml Leupotinin and100 nM NaF. The homogenate was centrifuged at 20,000 g for 10 minutes; the supernatant was retained, aliquoted and stored at -80°C. Protein concentration was determined by [27] using a commercial kit (Bio-Rad Laboratories; Hercules, CA, USA). The AMPK activity of supernatant homogenate muscle samples was determined by using the kinase assay Z-Lyte-Ser/thr 23 peptide, a specific assay for AMPK (α1/2-β1-γ1) based on fluorescence resonance energy transfer (FRET) (Invitrogen^™^, Life Technologies, PV4644), according to Apaoblaza et al., [5], with modifications.

In this assay, the Z´-LYTE^™^ Peptide Substrate functions as a 0% phosphorylation control for the assay, and the kit also contains a Z´-LYTE^™^ Phospho-peptide, which functions as a synthetic 100% phosphorylation control. These controls establish minimum and maximum emission ratio values on the assay plate and are used to convert experimental emission ratios to calculated percent phosphorylation values. A 20 μl peptide substrate Z-lyte 2X/ATP 2x in 5x kinase buffer was added to 20 μl sample diluted at 40 ug/ul in 50 mM 10 mM MgCl Hepes buffer pH 7.5. The solution was incubated for 1 h at room temperature (initial and final characterization of the samples). Subsequently, 20 μl of Z-Lyte development solution were added, and samples were incubated for 1 h at room temperature. Finally, 20 μl of stop reagent were added to each well and then the signals of coumarin and fluorescein emission (at 445 and 520 nm respectively) were measured using the Thermo Scientific Varioskan Flash reader.

### Effect of temperature on AMPK activity

For this experiment, treatments consisted in incubation of the samples (both high and low final pH) for 1h at four different temperatures, one close to physiologic condition (37°C) and three intermediate temperatures in their normal cooling rate to 0–2°C in cooling room (25°C, 17°C and 5°C), before addition of the stop agent and measuring of fluorescein.

### Effect of glycogen addition on AMPK activity

In order to evaluate the possible regulation of AMPK activity by available glycogen concentrations, supernatants of homogenised samples from both categories were supplemented with 60 mmol/kg of glycogen (corresponding to the differential mean concentration of glycogen between carcasses that had normal vs high pH). For this experiment, treatments consisted in the incubation of the samples (both categories) for 1h at 17°C without or with previous glycogen addition, before addition of the stop agent and measuring fluorescein. Only samples from biopsies obtained at 0.5 h postmortem were used.

### Glycogen type analysis

For this analysis, homogenates were processed according to Villarroel-Espíndola et al., [28]. For the structural study of muscle glycogen from the samples, the spectrophotometric assay described by Krishman [29] was used. Briefly, Iodine absorption spectra of glycogen are present in the homogenates of both category and time. Total glycogen was isolated from the respective samples by ethanol precipitation before spectrophotometric analysis. As a control, a commercial glucose polymer was used, such as muscle glycogen and purified amylopectin.

### Statistical analysis

All statistical analyses were conducted using a statistical package GraphPAD (Software of Science). The differences between means were determined using a two-way ANOVA followed by Bonferroni post-hoc test for multiple comparisons. Data are expressed as mean ± standard error for the mean (SEM). In all cases, a p ≤ 0.05 was considered as significant.

## Results

### Changes in pH, metabolic measure, and AMPK activity between categories

Changes in muscular pH, glycogen content, lactate accumulation, and AMPK activity at 0.5 and 24 h in carcasses from both pH categories are shown in Table 1. Regarding final pH reached by each group, the differences (p < 0.01) between high and low pH categories were near to 0.71 pH point (6.44 ± 0.05 vs 5.73 ± 0.03, respectively). Consistent with the final pH, the muscle lactate concentration at 24 h in the low pH group was 47% greater (p < 0.01) than in the high pH group (39.00±0.71 vs 57.43±2.15 mmol/Kg, respectively). On the other hand, the muscle glycogen concentration at 0.5 h postmortem in the low pH group was 365% greater (p < 0.01) than in the high pH group (16.55±0.87 vs 76.94±6.9 mmol/Kg, respectively). Finally, like in our previous study [5], the AMPK activity was significantly greater in the low pH group than in the high pH group at both 0.5 h (680% of difference, p < 0.01) and at 24 h (400% of difference, p < 0.01). The AMPK activity assays reported in this experiment were carried out at room temperature (17–20°C).

**Table 1:** Mean pH, muscle glycogen concentration (MGC), lactate (LA) and adenosine monophosphate kinase (AMPK) activities in *M*. *longissimus thoracis*, measured at 0.5 h (T0) and 24 h (T24) *postmortem* in steer carcasses categorized with normal (<5.8) or high (>5.9) final pH.

Variables	High pH	Normal pH	Two-way Anova (*P*—value)		
	(Mean ± SEM)	(Mean ± SEM)	pH	Time	pH x Time
pH (T0)	6.91±0.16^a^	6.73±0.16^a^	0.0001	< 0.0001	0.0099
pH (T24)	6.44±0.05^b^	5.73±0.03^c^			
MGC (T0) (mmol/Kg)	16.55±0.87^a, c^	76.94±6.9^b^	0.0001	< 0.0001	0.0004
MGC (T24) (mmol/Kg)	0.09±0.09^a^	23.24±5.27^c^			
LA (T0) (mmol/Kg)	31.20±2.84^a^	50.39±2.27^b^	< 0.0001	0.0041	NS
LA (T24) (mmol/Kg)	39.00±0.71^a^	57.43±2.15^b^			
AMPK (T0) (Δac)	2.87±1.03^a^	22.36±1.99^b^	< 0.0001	NS	NS
AMPK (T24) (Δac)	5.66±1.18^a^	28.39±7.06^b^			

Means in a row without a common superscript letter differ (P < 0.05) as analysed by two-way ANOVA and Bonferroni’s multiple comparisons.

### Temperature decline and changes in AMPK activity

**Fig 1:**
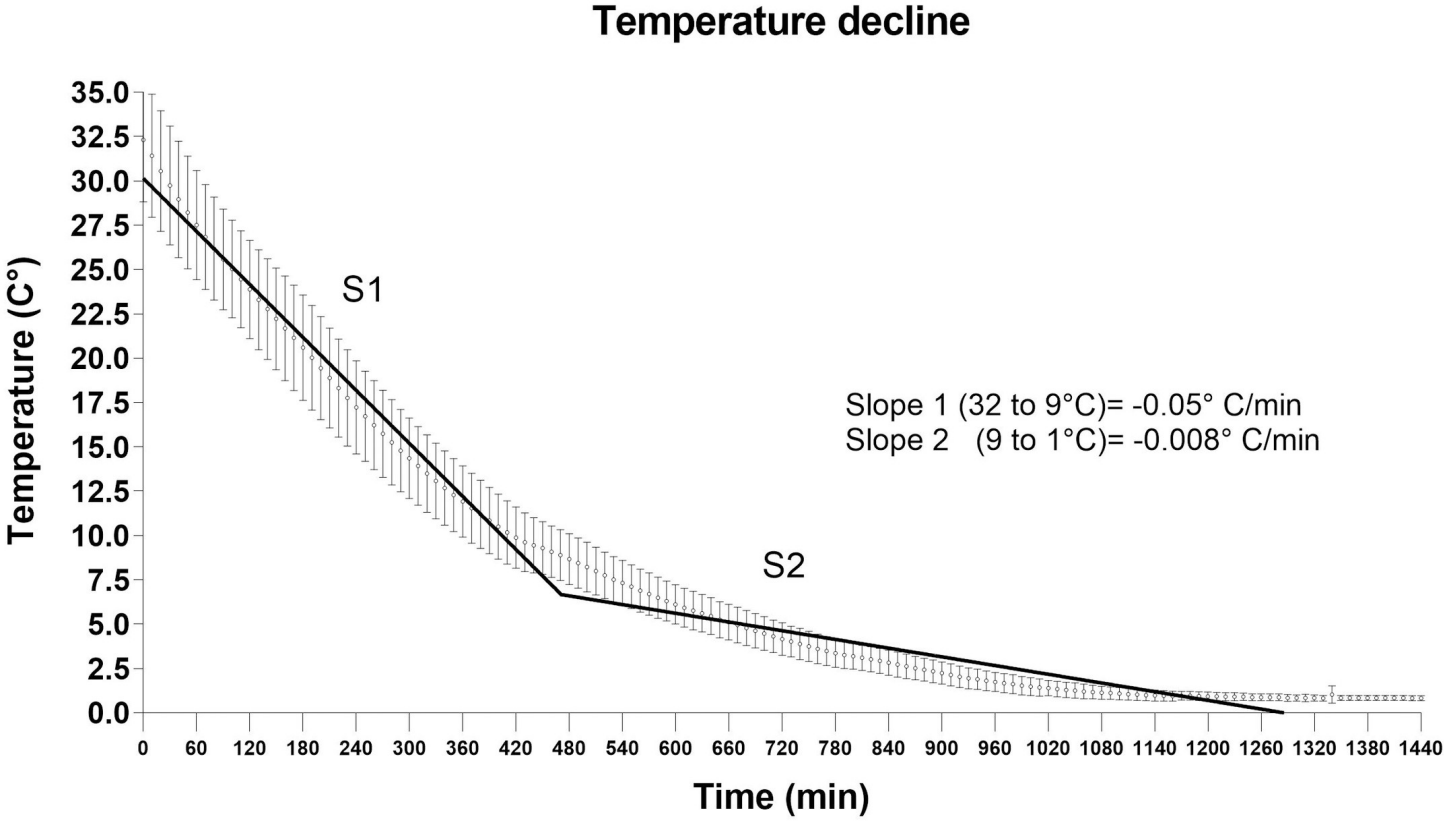
Temperature decline *postmortem* in steer carcasses *M*. *longissimus thoracis* (both categories, n = 12). In the figure, slopes are shown (S1 and S2) corresponding to two components founded by non-linear function analysis.

The cooling rate (0 to 24 h, in the cooling room), of all carcasses used is shown in Fig 1. Analysis of the obtained non-linear function identified two components (slopes), the first from 32° to 9°C (S1: -0.05°C/min) and the second from 9° to 1°C (S2: -0.008°C/min). Analyses of enzymatic activity of AMPK consistently showed a greater and sustained AMPK activity in muscle samples from carcasses categorized with normal final pH vs. those with high pH. Values were 680 and 400% at 0.5 and at 24 h postmortem, respectively (Table 1).

**Fig 2:**
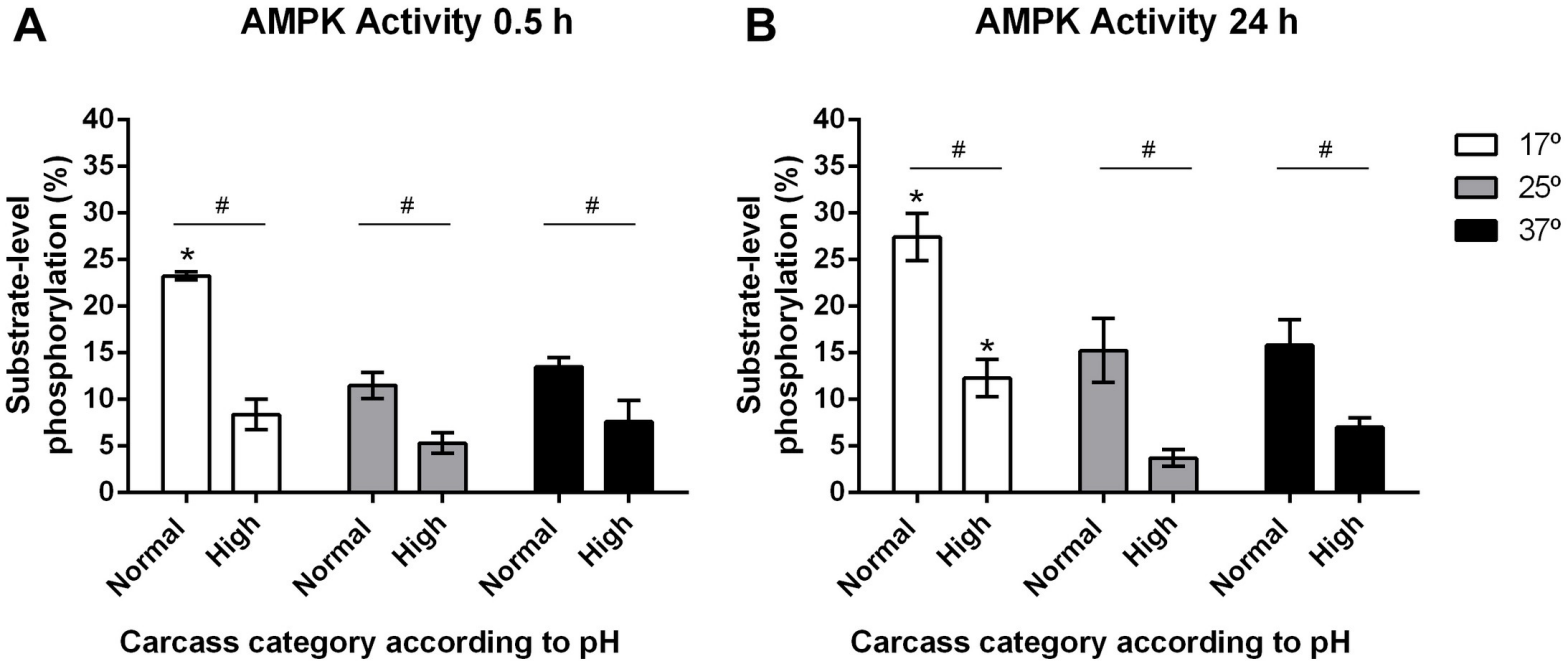
Average values ±SEM of AMPK enzyme activity levels in samples of in *M*. *longissimus thoracis* categorized with normal (< 5.8) v/s high (> 5.9) final pH, evaluated at 0.5 h and 24 h and in function of temperature. A: measure at 17, 25 and 37°C at the 0.5 h; B: measure at 17, 25 and 37°C at the 24 h. Two-way ANOVA and Bonferroni post-tests. Differences between categories (#, p < 0.0001) and between 17°C and 25°C or 37°C, within categories (*, p < 0.0001), n = 6.

The results of AMPK enzymatic activity analysis under three different temperatures, are shown in Fig 2 and the analysis of the samples taken at 0.5 h postmortem (T0) incubated at the three temperatures used confirmed a greater AMPK activity in the meat samples from carcasses that reached a normal final pH than those with high final pH (p <0.0001, Fig 2A).

The results showed that AMPK activity at 0.5 h postmortem did not vary according to temperature. The only exception was at 17°C (samples with normal final pH), where AMPK activity showed to be greater than at 25° and 37° (p <0.0001, Fig 2A), and even greater with respect to measurements obtained at 17°, 25° and 37°C in samples with high final pH (p <0.05, Fig 2B).

Comparative measurements at 24 h showed the same trend compared to 0.5 h, although less accentuated, with the novelty that average AMPK activity at 17°C was greater in the samples from carcasses with high pH than at 25 and 37°C, in the same category (p <0.05, Fig 2B).

To expand the study of the effect of temperature on the enzymatic activity of AMPK, a second experiment was performed with the inclusion of a thermal point closer to the final temperature of maintenance in the cooling room (0–2°C). According to our records, this temperature was reached between 11–12 h after the carcasses entered the cooling room (720 min on the x-axis, Fig 1). Considering that there were no differences associated with time (0.5 vs 24 h), only samples from biopsies taken at 0.5 h were analysed.

**Fig 3:**
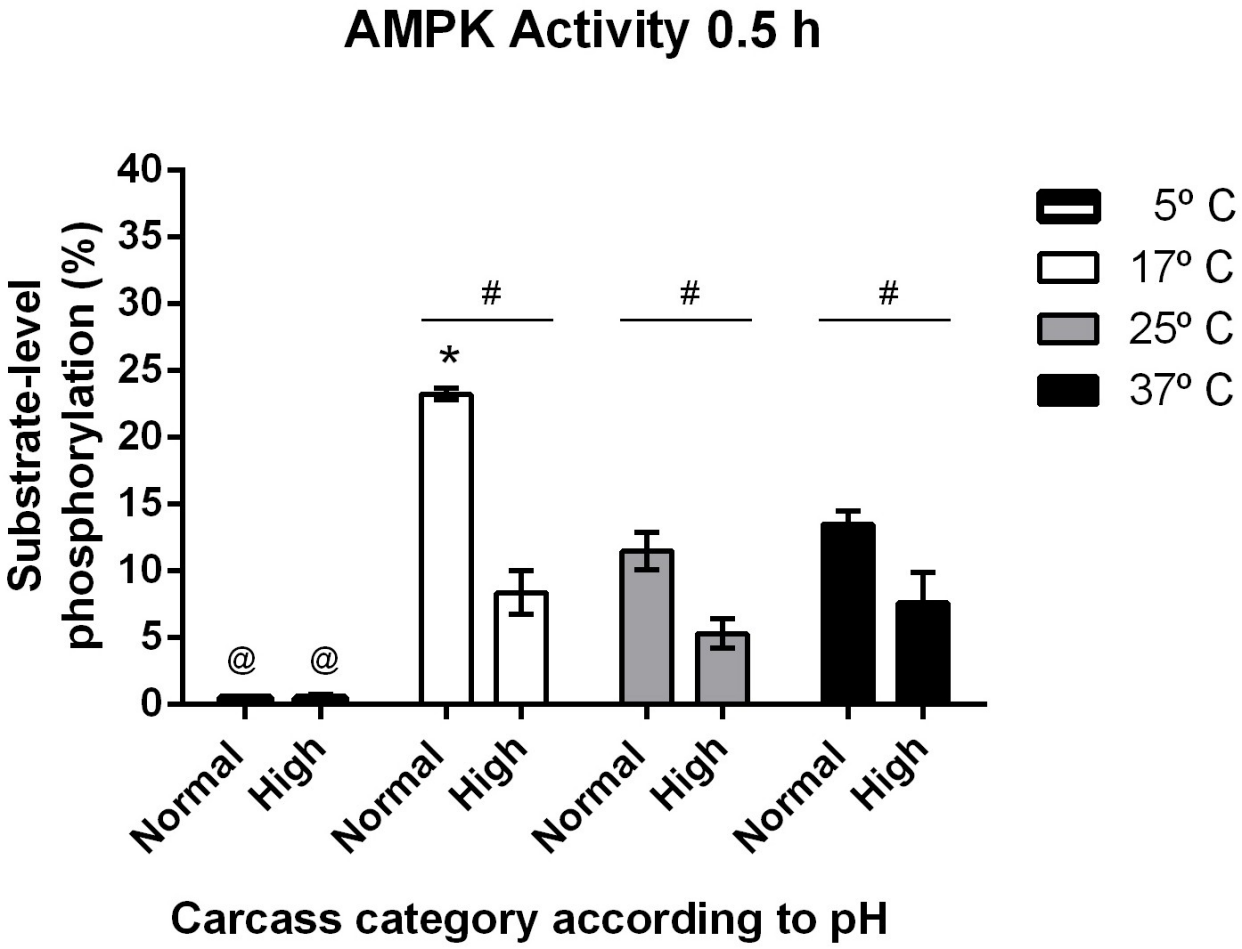
Average values ±SEM of AMPK enzyme activity levels in samples of in *M*. *longissimus thoracis* categorized with normal (< 5.8) v/s high (> 5.9) final pH evaluated at different temperatures in the cooling phase (5°, 17°, 25° and 37°C at the 0.5 h). Two-way ANOVA and Bonferroni post-tests. Differences between categories (#, p < 0.0001); between 17°C and 25°C or 37°C, within categories (*, p < 0.0001); between 17°C and 5°C, within categories (@. p < 0.0001 and p < 0.05, for normal and high final pH, respectively), n = 6.

The AMPK activity analysis at different temperatures (5, 17, 25 and 37°C) showed that the enzyme is strongly regulated for this factor. Effectively, in the samples from the low pH group and at 0.5 h, the AMPK activity was maximal at 17°C and was completely inhibited at 5°C (p < 0.01, Fig 3). In the high pH group, all measured AMPK activity level was lower than in the low pH group (p <0.01, Fig 3), and the activity the significant inhibited at 5°C (p <0.05, Fig 3).

### AMPK activity and glycogen content in postmortem muscle

**Fig 4:**
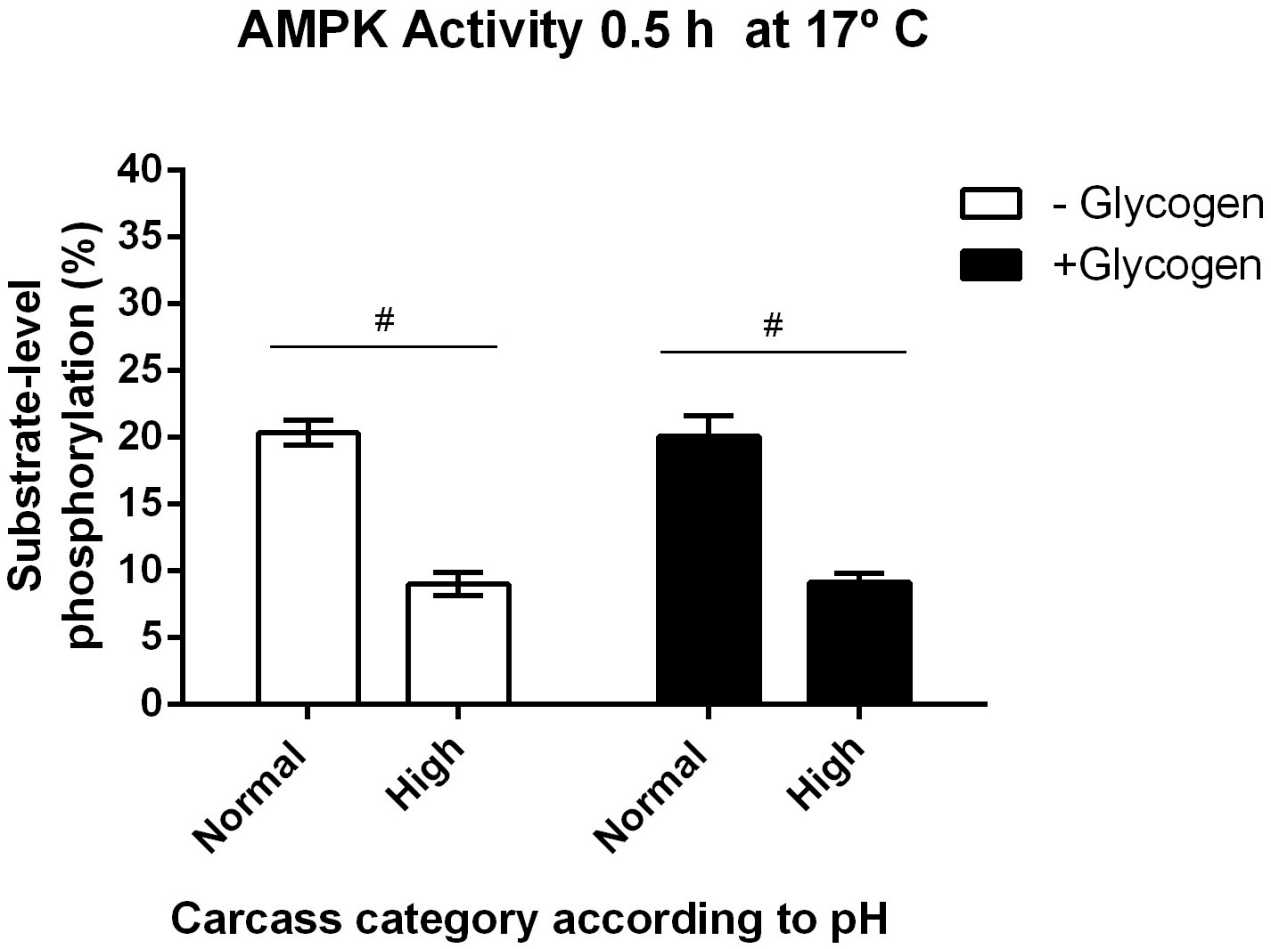
Average values ±SEM of AMPK enzyme activity levels in samples of *M*. *longissimus thoracis* categorized with normal (< 5.8) v/s high (> 5.9) final pH, evaluated at 0.5 h and to 17°C, and in function of endogen and exogenous glycogen content. Two-way ANOVA and Bonferroni post-tests. Differences between categories (#, p < 0.0001), n = 6.

The AMPK enzymatic assays for this study were measured at 17°C, which has showed the optimal condition for AMPK activity. Under these conditions, the concentrations of glycogen detected each sample were 76 (16 + 60) and 136 (76 + 60) mmol/kg of skeletal muscle from high and normal final pH, respectively. Results showed that AMPK activity was not affected by the naturally stored glycogen; however, exogenous added glycogen slightly reduced the activity. The pH seemed not to be a relevant factor to modulate AMPK enzymatic activity under the used experimental conditions (Fig 4).

As mentioned above, during *in vitro* glycogen supplementation (60mmol / kg), we did not observe an inhibitory effect on AMPK activity. When the supplementation was the double of the basal concentration of glycogen (136 mmol/kg), the activity of AMPK remained unchanged.

### Analysis of glycogen type

**Fig 5:**
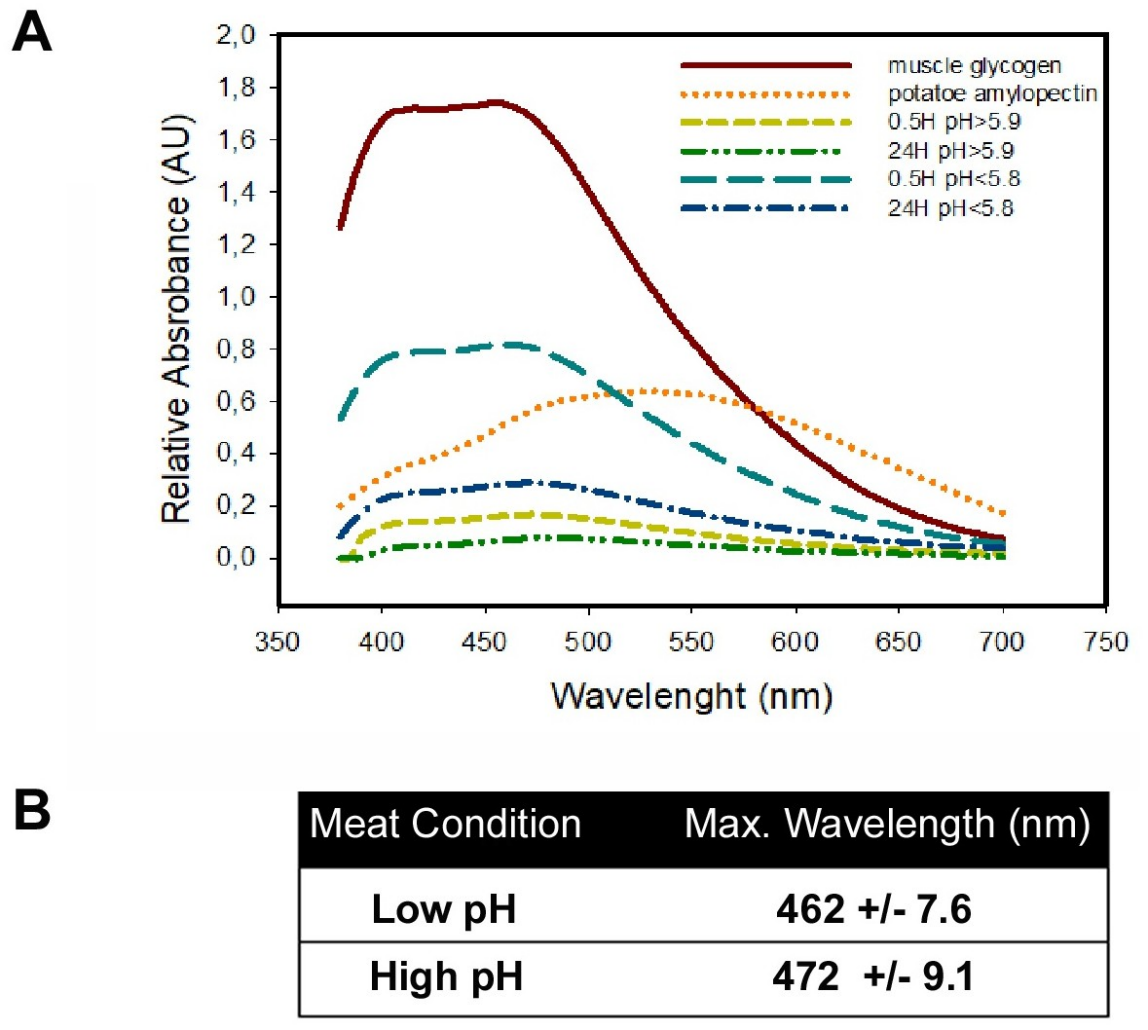
Structural glycogen analysis. A: Absorption spectrum of glycogen in samples of *M*. *longissimus thoracis* categorized with normal (< 5.8) v/s high (> 5.9) final pH, in presence of potassium iodide. B: maxima wavelength (nm). (n = 6).

To evaluate if the glycogen types in the samples presented structural differences, an analysis of the absorption spectrum of glycogen in the presence of iodine was carried out. Analyses showed that there were no differences in the glycogen types between samples from the low pH group and the high pH group (p> 0.05, Fig 5A and 5B). Moreover, the obtained wavelengths coincided with those described for muscle glycogen molecules in the presence of potassium iodide, confirming that there were no changes in the three-dimensional configuration of the glycogen molecule, keeping a stable degree of branching in samples from both pH groups in both conditions. This was evident when comparing the absorption spectrum of muscle glycogen purified by precipitation and the absorption profile characteristics of amylopectin, which have a lower degree of branching and have a displacement to the right of the maximum lambda, around 550 nm in Fig 5A.

The glycogen spectrum analysis confirmed that the muscle samples collected from carcasses with high final pH presented lower amounts of glycogen than the samples obtained from carcasses categorized with normal final pH (at both 0.5 and 24 h postmortem). (Shown as pea green and green lines vs light blue and blue lines, respectively in Fig 5A).

In addition, results confirmed that samples from the high final pH group, at 0.5 h, had low concentrations of glycogen, since by plotting each spectrum only noise was observed (pea green line in Fig 5A); and a delta of absorbance was close to 10% of glycogen control (brown line in Fig 5A). Samples taken at 24 h from carcasses with normal pH presented a remarkable reduction in the relative intensities of absorbance, with respect to their basal control (0.5 h) (light blue and blue lines, respectively, in Fig 5A).

## Discussion

It is known that AMPK is an enzyme, which is activated in response to several stressors. Recently, Genders et al., [30] reported in rat cardiomyocytes, a decrease in p-AMPK relative abundance in low pH with lactate conditions. Similarly, other authors showed that AMPK activity in cultured cardiomyocytes was suppressed upon acidic treatment but was stimulated upon alkaline treatment [31].

Contrarily to the above mentioned studies, in our study we showed a greater AMPK activity in muscle samples from steer carcasses with a low final pH than in those with a high final pH (630 and 400% greater at 0.5 and 24 h, respectively, Table 1). However, our group [5, 7] like those reports our results previously.

Moreover, we showed that the differences in pH between both categories started 120 minutes after the carcasses entered the cooling room; therefore the differences in AMPK activity between both pH categories started soon (before 30 minutes), even without being different in pH [32, 33]. On the other hand, AMPK activity did not appear to vary in samples from the low final pH group, in which the pH declined by 1 unit of pH in 24 h (6.73 to 5.73, Table 1), similar to those reported by our group in a previous study [5].

Temperature and other stressors associated with cooling or freezing (ischemia and osmotic shock) affect the function of several tissues and organs in animals [15–17, 34]. Regarding the AMPK activity regulation by environmental stressors both hypoxia and hypothermia in frogs increase AMPK phosphorylation level and AMPK activity [19]. Furthermore, in the hypothermia of the Rana sylvatica induced by freezing, AMPK enzyme increased their activity [20]. This suggests that AMPK activation *in vivo* would facilitate adaptation to hypothermia, freezing and thawing.

The decline in pH in skeletal muscles postmortem is due to the formation of lactate from stored glycogen [35]. Furthermore, temperature linearly affects the rate of decline in muscle pH in postmortem samples, from a body temperature down to 5°C, with a slower rate of decline at progressively lower temperatures [36].

The primary enzymes that regulate the rate of postmorterm glycolysis in muscle are glycogen phosphorylase (GP) and phosphofructokinase (PFK) [37]. Previous studies reported by Helmreich and Cori [38] in living muscle from frog and by Newbold and Scopes [39] in Ox muscle postmortem, showed that of the 12 enzyme reactions involved in the conversion of glycogen into lactate, both the phosphorylase and phosphofructokinase reactions are of main importance in the regulation of glycogen breakdown in both conditions (live or postmortem).

Newbold and Scopes [39], suggested that the activation of glycolysis below 5°C appears to be due to the greater accumulation of AMP which stimulates glycogenolysis by activating GP and stimulating PFK in postmortem muscle. On the other hand, AMP is also the main and direct activator of AMPK in postmortem muscle, and it has been shown that there is a strong correlation between AMPK activation and pH decline, indicating that AMPK regulates glycolysis in postmortem muscles [8, 21, 23]. Regulation of glycolysis by AMPK in postmortem muscle is mediated, at least partially, through phosphorylation and activation of PFK-2, since fructose-2,6-bisphosphate content, an allosteric activator of PFK-1, correlated well with AMPK activation and the glycolytic rate [40].

Few reports tackle postmortem changes in AMPK activity associated to low temperature exposures. However, carcass cooling can vary according to the slaughter plant, season, species, fat cover, carcass weight, and others process capacity, which can be relevant for meat quality. Studies in turkey muscles showed that a high temperature early postmortem could induce AMPK activation, which results in rapid glycolysis, thus affecting protein solubility and generating PSE characteristics [9].

During postmortem management of carcasses, the "physiologic context" of the muscle includes hypoxia and cooling. Regarding cooling, we showed that the carcasses in the cooling room experience two cooling rates: The first (32°C to 9°C) near to -0.05°/min; the second, (9° to 1°C), near to -0.015°C/min. This study and other studies showed that AMPK activity, measured between 17–20°C (room temperature) is four times greater in samples from carcasses with normal final pH than in carcasses with high final pH. For other hand, the level of the enzymatic activity (in both categories) is the same at early (0.5 h) and after 24 h postmortem [5] or is slightly greater [7].

Interestingly, the pH time-course in temperature function in this study shows that in the muscle samples maintained at 15°C, the ultimate pH (24-48-72 h) was lower than in those muscle samples kept at 1 or 5°C (near to 0.4 pH point at 24 h); and these differences begin at 7–8 h [39].

We believe that differences in pH between both categories could start earlier than 7 h, specifically closer to 3–4 h from the time that carcasses enter the cooling room, at the time where muscle temperature is near 20°C (Fig 1). Unpublished data by our group, based on records of pH kinetics, indicate that the carcasses reach 18°C 4 hours after entering the cooling room, time at which the difference in pH between the two groups is 0.5 pH points, and when the pH value of the normal pH carcasses is near to 6.

Based on our final pH results, and analyses derived from Newbold and Scopes [39], we argue that muscle samples from normal versus high pH carcasses experience a more efficient and consistent glycogenolytic and glycolytic flow, due to a higher initial glycogen reserve. The above, because the muscle samples already have a significant pH delta at 3–4 h, when the temperature is close to the optimal temperature for AMPK activity.

Comparatively, in the cooling room the temperature decline did not differ between the pH categories of carcasses analyzed (data no show, in Fig 1 shows only pooled data). However, it has been shown that the temperature decline of muscle in the cooling room can vary depending on anatomical location (i.e. deep v. superficial muscles), the weight and fatness of the carcass, and temperature and air-speed conditions during chilling [41]. Consequently, the glycolytic rate can vary not only between muscles, but also within a muscle [42].

In addition, Tarrant and Mothersill [42] showed that the rate of glycolysis in four beef muscles was, on average, 64% faster when the measurement was taken at a depth of 8 cm in the muscle compared with 5 cm, and the authors point out the importance of the relationship between pH and *in situ* temperature variation. For example, low pH values (<6.0) coincided with high temperatures (>30°C) in the muscles samples around 8 cm of depth. Moreover, combinations of low temperature (<10°C) and high pH (>6.0) were associated with cold shortening [42]. More recently, Hwang & Thompson [43] have estimated that the optimal rigor temperature could be higher than previously reported by *in vitro* studies, where it oscillated between 15–18°C [44, 45]. The optimum pH decline to produce the most tender meat after 14 days of age was achieved with a temperature of 29–30°C at pH 6, suggesting that these different results are based on differences between the constant temperature regimes used in the in vitro studies, and the declining temperature gradient in muscle samples *in situ* [46].

The results obtained by Tarrant and Mothersill [42] and our results, suggest that the temperature used in the cooling room can be a critical control point of postmortem glycogenolysis and glycolysis, and should allow the muscle piece (carcasses) to experience temperatures close to 15–17°C, at least for a few hours, and the carcasses should not be submitted to an accelerated cooling to 5°C, capable of neutralizing AMPK activity and its efficient control of glycogenolytic/glycolytic flux.

In addition to adenine nucleotides, glycogen and the non-physiological glycogen-mimic cyclodextrin may also regulate AMPK. However, while some authors have not detected an effect of glycogen on the activity of purified rat liver AMPK [47], other authors have reported that glycogen and cyclodextrin inhibit the catalytic activity of recombinant AMPK and native rat liver AMPK [48]. Similarly, it has been shown that glycogen regulation occurs both in presence and in absence of AMP and depends on its binding to the carbohydrate-binding loop of the CBM [49]. Interestingly, and related to AMPK/Glycogen interaction, McBride et al., [48], showed that glycogen inhibits purified AMPK in cell-free assays, by direct action of GBD, and that this varies according to the branching content of the glycogen. Moreover, these authors showed that the oligosaccharides used, are allosteric inhibitors of AMPK that also inhibit phosphorylation and activation by upstream kinases and suggested that the GBD is a regulatory domain that allows AMPK to act as a glycogen sensor in vivo. Like the report of Polekhina et al. [47], our results reveal that the enzymatic activity of AMPK was not affected by glycogen addition to the sample homogenate, in neither of the two pH categories used (Fig 4).

Results obtained after glycogen scavenging in the presence of iodide suggest that under conditions of high pH there is a lower basal amount of glycogen that drops close to 50% after 24 h. Under conditions of acidic pH, basal glycogen content is substantially higher than in alkaline conditions, being almost 4 times higher, declining to 75% in 24 h. In both conditions, there would be no abnormality in the type of polymer or the degree of ramifications thereof, behaving as conventional glycogen and not as amylopectin. This suggests that phosphorylase and debranching activity operated correctly during the *rigor mortis* process and that the observed muscle pH changes would not modulate these activities.

Our results give evidence that the postmortem enzymatic activity of AMPK is conditioned by the metabolic context and signal transduction present prior to animal slaughter. Other studies and using regression analysis (lean meat colour and pH at 24 h) concluded that cattle with lower pH at 1 h postmortem had a faster decline in pH and resulted in lower pH at 24 h [50]. Moreover, the same authors indicated that the high pH in cattle carcasses (i.e., Dark Firm Dry condition or DFD) were the result of their own initial potential prior to slaughter. Effectively, the mean of pH registered at three hours by Park et al. [50] were 6.6 and 6.0 (0.6 pH point of difference) for DFD and normal condition, respectively. On the other hand, in our study the mean initial pH values at 0.5 h postmortem were 6.91 and 6.73 (0.18 pH point) in the high and normal pH categories, respectively. This difference is slightly greater than that recorded in the study of Apaoblaza et al. [5] with 6.87 and 6.76 (0.11 pH point of difference) at 0.5 h and for the same categories.

Here, we report that the maximal AMPK activity in postmortem mammalian muscle occurred close to 17°C but not at 25°C or 37°C, and that AMPK activity is close to zero at 5°C. Interestingly, AMPK activity has been suggested as an adaptation to hypothermia in animals evolutionarily remote. Effectively, hypothermia in frogs increases the AMPK phosphorylation levels and AMPK activity [19, 20]. Moreover, in sperm cells, we have evaluated if pharmacological activation of AMPK improves the freezability of spermatozoa in stallions [51] and showed that the AMPK in this cellular model did not respond to classical activators such as AMP, AICAR and metformin. We found that the AMPK activity in stallion spermatozoa was 52% higher at 17°C than at 5°C (data not published) and we believe that the high cooling rates used in the study of Cordova et al., [51] between 20°C to 5°C were able to neutralize AMPK’s activity. Other authors have reported that porcine sperm stored at 17°C (optimal temperature for commercial use of pig semen for several days) increased AMPK activity, longevity, and sperm motility maintenance [52]. Thus, we postulate that regulation of AMPK activity by temperature might represent a control mechanism not restricted to postmortem muscle metabolism, but also to the *in vivo* muscle metabolism and other tissues and cells.

Thermo-sensibility and thermo-stability of AMPK activity in muscle and other tissues and cellular types is a field of study that can open interesting windows for improving the cooling managing of postmortem muscle and in other possible applications as organ transplants and cell preservation.

## Conclusions

AMPK activity is highly sensitive to temperature, presenting maximum levels of activity at 17°C and minimal levels at 5°C. The *in vitro* excess of glycogen does not affect the enzymatic activity of AMPK in postmortem cattle muscle. Normal concentrations of pre-mortem muscle glycogen and AMPK activity determine the efficient glycogenolytic/glycolytic flow required for lactate accumulation and pH decline.

## Abbreviations

AICAR: 5-Aminoimidazole-4-carboxamide ribonucleotide
AMP: Adenosine monophosphate
AMPK: AMP-activated protein kinase
ATP: Adenosine triphosphate
CBM: Carbohydrate-binding module
DFD: Dark Firm Dry
FRET: Fluorescence resonance energy transfer
GBD: Glycogen-binding domain
GP: Glycogen phosphorylase
LA: Lactate
MGC: Muscle glycogen muscle
PFK: Phosphofructokinase
PSE: Pale, soft and exudative
T0: Temperature at 0–30 minutes
T24: Temperature at 24 h
